# Health professionals’ knowledge about relative prevalence of hospital-acquired infections in Delta State of Nigeria

**DOI:** 10.11604/pamj.2016.24.148.9270

**Published:** 2016-06-20

**Authors:** Angus Nnamdi Oli, Kelechi Christian Okoli, Nonye Treasure Ujam, Dave Ufuoma Adje, Ifeanyi Ezeobi

**Affiliations:** 1 Department of Pharmaceutical Microbiology and Biotechnology, Faculty of Pharmaceutical Sciences, Nnamdi Azikiwe University, Agulu, Anambra State, Nigeria; 2 Department of Clinical Pharmacy and Pharmacy Administration, Faculty of Pharmacy, Delta State University, Abraka, Nigeria; 3 Department of Orthopedic Surgery, Chukwuemeka Odumegwu Ojukwu University Teaching Hospital Amaku-Awka, Anambra State, Nigeria

**Keywords:** Nosocomial infections, prevalence, knowledge, infections control, Delta

## Abstract

**Introduction:**

Hospital-acquired infections (HAIs) constitute a serious global public health challenge, causing great suffering to many people across the globe at any given time. This study ascertains the knowledge of health professionals on the challenge and their compliance with infection control measures.

**Methods:**

Validated questionnaires were administered to 660 health professionals and supported with face-to-face interview. Data were analyzed using Statistical Package for Social Sciences (SPSS) version 16.0 (SPSS Inc, USA). Chi-square was used to test association between the independent and the outcome variables. Cut-off point for statistical significance was 5% (p value<0.05).

**Results:**

UTIs (61.4%) followed by Hospital-acquired Pneumonia (55.6%) were known to be the most prevalent HAIs in government hospitals while *Staphylococcus aureus* (54.4%) was reported the most microbial agent. In private health facilities, Hospital-acquired Pneumonia was known to be the most common (66.1%) while *Pseudomonas aeruginosa* was the most reported culprit. HAIs were reported to have occurred more in government hospitals and catheterization was the commonest modes of transmission in both health facilities.

**Conclusion:**

The prevalence of HAIs in this state was reported to be high. Although health-care professionals have good knowledge of HAIs, active effort is not always made to identify and resolve them. Standardized surveillance of HAIs is urgently needed.

## Introduction

The primary purpose of hospitals is to ensure that sick people recover from their illnesses but this is not always so. Sometimes, illnesses get complicated and healthy people get infected in healthcare facilities. The occurrence and unwanted consequences of nosocomial infections have been known for several decades and continue to escalate at an alarming rate [1]. HAIs constitute a serious global public health challenge, causing untold suffering to about 1.4 million people across the world at any given time [2]. They often increase costs of health care on both patients and health services [3]. Many HAIs are preventable as it has been shown that compliance with guidelines greatly reduces both the rate and number of infections [4].

Among the many factors responsible for continued increase of HAIs in hospitalized patients are: poor immune status of patients; extremes of age, use of medical procedures and/or invasive techniques/devices, emergence of drug-resistant bacteria and over-crowding in hospitals [5–7]. Poor infection control practices may also facilitate transmission. Studies found that hand washing, education, breast-feeding, proper food preparation, personal hygiene, knowledge of risky practices, immunization, interaction with public health officials when illness occurs and interruption of fecal-oral spread [7–9] are all essential for containment of HAIs.

HAIs affect both developed and developing countries and constitute “major causes of death and increased morbidity among hospitalized patients” [10]. The most frequently encountered HAIs are urinary tract infections (UTI), infections of surgical wound and lower respiratory tract infections [11, 12]. Hospital-acquired UTI has been shown to contribute immensely to UTI in some parts of northern Nigeria, causing about 43% of the UTIs [13].

A good knowledge of health professional about HAIs will help reduce their prevalence among hospitalized patient and the health workers [14, 15]. In Delta state, it is not known the extent the state is affected by the health issue. Although the state had implemented and is still implementing several health programmes [16], it has not yet instituted HAIs surveillance/control programme and has not seen such as a Gaps in Performance. Assessing compliance of the health-care professionals in the state with infection control measures is also vital. The study therefore examined the burden of nosocomial infection in Delta State of Nigeria, the knowledge of healthcare workers in the State to the problem and their compliance with infection control measures.

## Methods

Structured questionnaires were administered to study participants selected purposively from health facilities located in 15 Local Government Areas in Delta State. Correct definition of nosocomial infection was used as a yardstick for ruling out possibility of confounding effect.

### Study area

The study was carried out in Delta state of Nigeria. The State is ethnically diverse and seven major languages and dialects are indigenous in the state. The major ethnic groups in the state are: Ijaw, Urhobo, Itshekiri, Isoko, and Kwale/Anioma. The 25 Local Government Areas (LGAs) in the state are grouped under Delta North, Delta South and Delta Central senatorial districts. The capital city is Asaba while Warri is the biggest commercial city in the state.

### Study participants

The participants in this study included: medical doctors, pharmacists, nurses and the medical laboratory scientists. These professionals were chosen proportionally based on their population in the state. They were chosen because they were assumed to have better knowledge of the infections than others due to their practice and training.

Instrument for data collection and sampling method: Modified pre-validated questionnaires [17], were used as the main instrument for data collection. The questionnaires were pretested in a tertiary hospital and necessary corrections made before administering to the study participants. Assessment of the prevalence of HAIs was done via questionnaire and direct interview to find out the infections the study participants had encountered in their practices while the knowledge of HAIs and control practice was assessed by questions relating to the knowledge/definition of HAIs and practice of the core elements of universal precaution.

Study participants were selected using multi-stage sampling technique. Out of the 25 LGAs, 15 were purposively selected. The health facilities visited in the LGAs were also chosen purposively. Questionnaires were administered to the participants working in: the theatre, intensive care unit, the medical, surgical and/or gyneacology, neonatal and/or peadiatrics wards, chemical pathology, bacteriology and/or parasitology, blood bank and/or hematology, histopathology laboratories, the HIV care unit and the compounding and/or dispensing pharmacy units and who were present at the time of visit. These units were chosen because of the observed higher tendency of encountering HAIs in them.

### Data analysis

Data was analyzed using SPSS version 16.0 (SPSS Inc, USA). The proportion of practioners in government hospitals with knowledge on HAIs was compared to their counterparts in private establishments using chi-square. Cut-off point for statistical significance was set at 5% (p value<0.05).

## Results

Out of the 660 study participants 96 were doctors, 170 were nurses, 24 were pharmacists and 40 were medical laboratory scientists from selected government health facilities and while in private hospitals 90 were doctors, 180 were nurses, 30 were pharmacists and 30 were medical laboratory scientists. The most commonly encountered HAIs in the government hospitals were Urinary Tract Infections (UTI) (61.4%) followed by hospital-acquired pneumonia (55.6%) while the least were Ventilator-associated pneumonia (1.9%). In the private health facilities, hospital-acquired pneumonia was the most prevalent (66.1%) and closely followed by UTI (62.4%) while the least was Tuberculosis (1.8%). There was a significant difference in the prevalence rate of HAIs between government and private health facilities with the infection occurring more in the government hospitals, may be due to overcrowding (Chi-square = 134.0, P value < 0.0001) (Table 1).

**Table 1:** Awareness of occurrence of Hospital Acquired Infections (HAIs)

Hospital-Acquired Infections (HAIs)	Percent occurrences in Government hospitals (%)					Percent occurrences in private hospitals (%)				
	H	L	M	NI	N	H	L	M	NI	N
Ventilator associated pneumonia	1.9	18.9	40.0	25.7	13.6	13.9	15.2	55.8	6.1	9.1
Tuberculosis	17.7	14.1	51.6	7.20	9.9	1.8	25.5	67.9	4.8	0.0
Hospital acquired pneumonia	55.6	20.1	22.6	1.7	0.0	66.1	7.3	25.5	0.0	1.2
Gastroenteritis	52.7	22.1	17.7	6.9	0.7	43.6	17.6	30.9	4.2	3.6
UTI	61.4	61.4	28.8	0.0	0.0	62.4	3.6	32.7	0.0	1.2
Surgical Infections	10.6	21.1	19.9	43.1	5.3	0.0	29.1	15.8	53.3	1.8
Fungi infections	16.8	48.1	21.6	10.6	2.2	4.2	25.5	21.8	42.4	6.1

Key: High (**H**) = > 10 cases in 1 year, Moderate (**M**) = 8 - 10 cases in 1 year, Low (**L**) = 5 - 7 cases in 1 year, Occasional; (N) = 1 - 4 cases in 1 year and NI = no idea

*Staphylococcus aureus* was the most encountered causative agent in government hospitals (54.4%) distantly followed by *Candida albicans/Aspergillus* (16.8%). Methicillin/Vancomycin Resistant Bacteria were least encountered (10.6%). *Pseudomonas aeruginosa* was the most prevalent causative agent in private health facilities (13.3%) followed by *Staphylococcus aureus* (4.8%) while *Mycobacterium Tuberculosis* was the least encountered (1.8%). The causative agents were found more in the government hospitals (Chi-square = 89.53, P value < 0.0001) (Table 2).

**Table 2:** Frequency of occurrence of the etiological agents

Etiological agents	Percent occurrences in Government hospitals (%)					Percent occurrences in private hospitals (%)				
	H	L	M	NI	N	H	L	M	NI	N
*Staphylococcus aureus*	54.4	14.9	17.5	7.9	5.3	4.8	31.5	58.2	4.2	1.2
*Clostridium difficile*	12.5	51.6	18.2	12.2	5.5	4.8	59.4	17.0	11.5	7.3
*Mycobacterium Tuberculosis*	17.7	14.1	51.6	7.20	9.9	1.8	25.5	67.9	4.8	0.0
Methicillin/ Vancomycin Resistant Bacteria	10.6	21.1	19.9	43.1	5.3	0.0	29.1	15.8	53.3	1.8
*Candida albicans/ Aspergillus*	16.8	48.1	21.6	10.6	2.2	4.2	25.5	21.8	42.4	6.1
*Pseudomonas aeruginosa*	12.5	17.3	55.2	9.6	5.3	13.3	52.7	27.9	0.0	6.1

Key: High (**H**) = > 10 cases in 1 year, Moderate (**M**) = 8 - 10 cases in 1 year, Low (**L**) = 5 - 7 cases in 1 year, Occasional; (N) = 1 - 4 cases in 1 year and NI = no idea

The commonest mode of transmission of HAIs was catheterization (96.4% and 86.7% in government and private health facilities respectively). Contact with blood and body fluid was the second most common HAIs transmission mode, followed closely by transmission via contaminated hands (89.7% and 65.45% respectively). Needle pricks and contaminated instruments were reported to be the least mode of transmission. A slight significant difference exists between both divisions of health practitioners on the modes of transmission of HAIs (Chi-square = 11.21, P value = 0.0474) (Table 3).

**Table 3:** Knowledge of modes of transmission of HAI

Modes of HAI transmission	Government n (%)	Private n (%)
Catheterization	318 (96.36)	286 (86.67)
Contact with blood / body fluid	310 (93.94)	262 (79.39)
Contaminated hands	296 (89.70)	216 (65.45)
Airborne	277 (83.94)	198 (60.00)
Needle Prick	165 (50)	165 (50)
Contaminated instruments	165 (50)	165 (50)

Personal Protective Equipment (PPE) and hand hygiene were the most frequently used preventive measures for HAIs in the state. Participants demonstrated good knowledge of HAIs preventive measures. Government workers significantly had better knowledge of preventive measures than private individuals (Chi-square, 28.88, p value = 0.0001). All the respondents indicated that they washed their hands to prevent cross infection but a very low percentage of them (0% from government and 6.1% from private) indicated that they are motivated to wash their hands to prevent HIV/Hepatitis B infection (Table 4).

**Table 4:** The knowledge of preventive measures to HAIs and reasons for hand hygiene

Knowledge of preventive measures to HAIs		
Methods of HAI prevention	Government n (%)	Private n (%)
Hand hygiene	313 (94.84)	248 (75.15)
Use of PPE	324 (98.18)	298 (90.30)
Proper disposal of medical waste	277 (83.94)	254 (76.97)
Processing of instruments	183 (55.45)	238 (72.12)
Isolation of Patients	205 (62.12)	124 (37.58)
**Reasons for hand hygiene**		
Prevent cross injection	330 (100)	330 (100)
Availability of soap & water	158 (48.0)	94 (28.5)
Patient's appearance	174 (52.8)	138 (41.8)
Sake of HIV/Hepatitis B	0	20 (6.1)

Table 5 shows the list of Personal Protective Equipment used by the study participants. All the respondents claimed to use hand-gloves while most used laboratory/clinical coats. The participants that used face masks were mostly surgeons and theater personnel. Health professionals from government hospitals always wore personal protective equipment (100%) as contrasted with those in private facilities where lapses were observed in the use of PPE (95.76%)

**Table 5:** List of Personal Protective Equipment (PPE) in use and frequency of use

List of Personal Protective Equipment (PPE) in use		
PPE	Government n (%)	Private n (%)
Hand gloves	330 (100)	330 (100)
Lab/clinical coat	328 (99.39)	296 (89.70)
Cover Shoes	154 (46.8)	124 (37.6)
Face mask	202 (61.1)	142 (43.0)
Goggles	52 (15.8)	20 (6.1)
Cap	24 (7.2)	12 (3.6)
**Frequency of use**		
Always	330 (100)	316 (95.76)
Sometimes	0	14 (4.24)
Rarely	0	0
Never	0	0

Table 6 shows that re-use of syringes is still practiced in some hospitals, although the number is insignificant. Up to 99% of the workers never re-use syringes and needles. Greater number of the health professionals in government hospitals disposes their used syringes/needles into puncture proof boxes while more respondents in private health facilities disposed used-syringes/needles into waste cartons and cans. Best practice in infection control is therefore not strictly followed in all the private health facilities visited.

**Table 6:** Frequency of syringes/needles reuse/disposal of used syringes and needles

Frequency of syringes/needles reuse		
Frequency	Government n (%)	Private n (%)
Always	0	0
Sometimes	3 (1)	4 (1.2)
Really	0	0
Never	327 (99)	326 (98.8)
**Disposal of used syringes and needles**		
Methods of used syringe disposal	**Government n (%)**	**Private n (%)**
Decontaminate before disposal	0	0
Dispose straight into puncture proof boxes	240 (72.6)	148 (44.8)
Dispose straight into waste carton/cans	90 (27.4)	182 (55.2)

Many workers in private are not aware if Infection Control Committee exists in their health facilities (Table 7). Some workers in the private were emphatic that the Committee was not in existence. A better picture was seen in the government hospitals. There, a significantly greater number of the workers asserted that the committee exists while only very few said that they had no idea.

**Table 7:** Existence of Infection Control Committee

Response	Government n (%)	Private n (%)
Yes	298 (90.30)	145 (43.93)
No	0	34 (10.30)
No idea	32 (9.70)	151 (45.76)

## Discussion

This study revealed that HAIs are common in both government and private facilities in the state. UTIs were the commonest HAIs in government, (61.4%) and private health facilities (62.4%). This might be due to the high usage of indwelling urinary catheters in hospitals. Nosocomial UTI had been reported as the most common complication in surgical patients [18]. In a study at Ronald Ross General Hospital Zambia, pneumonia was reported to be the commonest HAI encountered; the second being UTI (62.4%) [19]. The prevalence rate of UTI as reported by study participants in this study is higher than the 22% recorded by Ekweozor and Onyemenen [20] in Ibadan, the 38.6% rate recorded by Akinyemi et al [21] in Lagos and 35.5% rate recorded by Ebie *et al* [22] in Rukuba Military Cantonment, Jos (all in Nigeria) but lower than the 77.9% rate reported by Mbata [23] among prison inmates in Nigeria. The higher prevalence of HAI in public hospitals has been linked to the higher population of patients which cause overcrowding [6, 12]. The high level of awareness of HAIs among health professionals observed in this study is similar to the results obtained by Adebimpe et al [24] in Oshogbo, south western and Oli et al [25] in south eastern Nigeria.

This study also showed that *staphylococcus aureus* (54.4%) and *pseudomonas aeruginosa* (13.3%) were the most implicated etiology of HAIs in government and private health facilities respectively. This is similar to results of a USA study [26]. *Staphylococcus aureus* is a ubiquitous bacteria existing both as a normal skin flora and a pathogen in human host. Being a skin microbiota, it can easily get access into body orifices, establish there and may become pathogenic. It‘s remarkable adaptability and versatility has prided it as one of the most endemic pathogen in clinical settings [27, 28]. Methicillin-resistant *S. aureus* (MRSA) has been proven to be one of the most worldwide spread nosocomial pathogen of the 20th century [29]. Other researchers [30, 31] have also reported *Staphylococcus aureus* as a common cause of HAIs in Nigeria. *Pseudomonas aeruginosa* infection is common in hospitalized patients. Although an opportunistic disease causing bacteria, it was reported to be the second most common etiology of nosocomial pneumonia in the USA, the third most common isolate in UTI and surgical site infections, and the fifth most common isolate in all sites of nosocomial infection [26]. A little less than half of the respondents in government hospitals and more than half in private health facilities had no idea about Methicillin/Vancomycin Resistant bacteria being causative agents of HAIs (Table 2). Several factors, ranging from patient factors to personnel and management factors contribute to the transmission of these infections. Our study (Table 3) showed that these infections were transmitted most via the use of catheters. Reports [18, 32] have shown that indwelling catheters was the most common means of transmitting nosocomial UTI in hospitalized patients. Urethral catheters introduce organisms into the bladder, promote urethral colonization by microorganisms and cause mucosal irritation. The high percentage of the professionals indicating that contaminated hands was a means of transmission of nosocomial infections might be an indication of the poor hand hygiene practice in these hospitals possibly because running water may not always be available. Hand hygiene is the responsibility of both the individual practitioner and the institution where they work. Air pollution and poor environmental hygiene also has some role to play in the transmission of these infections. Overcrowding, which is associated with poor environmental hygiene, has been blamed as one of the possible causes of high prevalence HAIs in government hospitals in Nigeria [12]. The respondents’ knowledge of Isolation as a means of preventing HAIs transmission is poor especially for the private health facilities. Government workers significantly had better knowledge of HAIs prevention than private individuals (Chi-square, 28.88, P value < 0.0001). Poor implementation of isolation might be due to inadequate spaces in most of our hospitals, although, Lynch [33], reported that the prevention and control of HAIs is cost effective and achievable even in a resource limited setting. Ayliffe *et al* [34] reported that isolation is an effective way of preventing the spread of HAIs amongst hospitalized patients even though it has proved to be highly discriminatory. Poor attention to hygiene, overcrowding, lack of an effective Infection Control (IC) program and shortage of trained IC providers had been reported as contributing factors to the transmission of HAIs [35, 36].

The most common means of HAIs prevention known to the respondents was the use of PPE (Table 4). HAIs may be linked to healthcare personnel unknowingly passing on infection to their susceptible patients. PPE helps prevent contact with an infectious agent or body fluid that may contain an infectious agent. Hand hygiene is very essential in minimizing HAIs transmission and should be practiced as a precaution whenever there is any patient contact. In our study, the motivation for hand hygiene among the personnel was mostly to prevent cross infection. Yet, more than half of the workers in government facilities and almost half in private facilities wear gloves for cosmetic reasons. Promotion of hand hygiene adherence in health care facilities is a priority and requires leadership, administrative support and financial resources [2].

Practitioners must protect themselves and their patients through regular and proper use of personal protective equipment (PPE) whenever on duty. All the respondents in the government and 95.8% from private health facilities always wore PPE (Table 5). PPE adherence among the respondents in the two settings showed a significant difference (p value = 0.001). The 95.8% reported from Ronald Ross General Hospitals, Zambia [19] correlates with our finding in private health facilities. The commonest PPE in use in the hospitals were hand gloves and laboratory coats while cap was the least. Majority of respondents in both government and private health facilities indicated that they never re-use syringe/needles (Table 6). This is good practice and complies with Safe Injection Practices [37]. A hundred percent compliance with safe injection practices was also recorded by Katowa et al [19]. There is high level of HIV/AIDS awareness in the country [38] and the link between HIV, Hepatitis C and B transmission and use of reused syringes has been established [39]. Safe disposal of used syringes/needles and other sharps help in minimizing the risk of exposure to HAIs. If not disposed of safely they can injure people and spread pathogens such as Hepatitis B and C viruses and HIV. Although there were no significant difference in the methods of disposal of used syringes/needles among the government and private health professionals (p value = 1) a greater percentage of the government employees (72.6%) dispose their used syringes/needles straight into puncture proof boxes, a lower percentage of the private employees (55.2%) make use of waste cartons/cans (Table 6) Use of any other disposal method for used syringes/needle except puncture proof boxes is unsafe and could encourage the spread of HALs. Non-availability of puncture proof boxes at all times may be the reason for using waste carton cans in some health facilities.

The Infection Control Committee is a critical component of infection control in hospitals. Where it exists, it helps to improve hospital infection control practice, and contributes to reduction in the rate of emergence of HAIs. It also recommends appropriate policies, which are subject to frequent review, by the same committee, depending on the prevailing circumstances in the hospital [40]. This study (Table 7) showed that this committee exists in most government hospitals but is lacking in more than half of the private health facilities. Also, nearly half of the respondents from private health facilities have no idea whether the committee exists or not. The lack of infection control committee in most private health facilities is a serious cause for concern and calls for urgent remedial action.

## Conclusion

The prevalence of nosocomial infection in this state was reported to be high. Although health-care workers have good knowledge of HAIs, active effort is not always made to identify and resolve the health challenge. Standardized surveillance of nosocomial infections is urgently needed in order to reduce nosocomial infections and improve the quality of life of patients and healthcare workers as well as healthcare delivery system.

### What is known about this topic

- HAIs cause morbidity, mortality and prolonged hospital admissions;
- Surveillance of HAIs in developing countries is relatively poor compared to developed economies;
- Good and regular surveillance systems reduces the burden.

### What this study adds

- Documentation of the knowledge of health professionals in the State on the challenge;
- Documentation of their compliance with infection control measures;
- The need for Standardized surveillance of HAIs in the State.

### Ethical approval and Compliance with Ethical Standards

Ethical permit/clearance was obtained from the Ministry of Health of the state (Approval Number: HD92/A2/37). This study followed every necessary international, national, and/or institutional ethical guideline and obeyed the 1964 Helsinki declaration and its later amendments or comparable ethical standards.

### Funding

The study was self-sponsored. No grant was secured
